# The Bascom Cleft Lift as a Solution for All Presentations of Pilonidal Disease

**DOI:** 10.7759/cureus.13053

**Published:** 2021-02-01

**Authors:** Steven C Immerman

**Affiliations:** 1 General Surgery, Evergreen Surgical, Eau Claire, USA

**Keywords:** pilonidal, pilonidal cyst surgery, pilonidal sinus surgery, cleft lift surgery, bascom cleft lift, pilonidal surgery, perianal pilonidal wounds

## Abstract

Objective

This study is a report on 700 consecutive patients treated with the Bascom cleft lift as treatment for both simple and complex pilonidal diseases between 1993 and 2020.

Patients and methods

The Bascom cleft lift was used in patients with primary disease, recurrent disease, perianal disease, and failed flap procedures. Some technical modifications had to be made to accommodate patients with perianal disease, and they are described. All patients were treated by the author in private practice clinics and hospitals between 1993 and 2020 and were entered into a database concomitantly with their treatment. Patients were subsequently surveyed in preparation for this study, by phone, email, or email survey to determine the current status. The procedure consisted of excision of the sinus tracts, cysts, and open wounds; raising a skin and subcutaneous tissue flap; and flattening the gluteal cleft. Failure of the procedure was defined as lack of complete healing or recurrent pilonidal disease requiring revisional surgery. Complications were recorded and are described.

Results

We found that of the 700 patients, 3.4% required revision of the cleft lift (confidence interval for proportion based on binomial distribution: 2.1%-4.8%). The median follow-up time for patients without recurrence was six months (IQR: 2-19.5). Once primary healing was obtained, there were no late recurrences in the 156 patients followed beyond 24 months. Factors that negatively impacted the success rate were having had previous failed pilonidal surgery (5.3% required revision) and open wounds on the edge of the anus (15.5% required revision).

Conclusions

The Bascom cleft lift had an overall success rate of 96.6%. There was no category of patients for which this was not a good option, but analysis of this data reveals that patients who have had previous failed surgery, and patients with wounds on the edge of the anus had a higher failure rate than the group as a whole.

## Introduction

Pilonidal disease is a complex problem for which many surgical approaches have been described for treatment. Part of the difficulty with the initial development of successful surgical strategies was that the etiology was ascribed to the presence of a congenital “cyst”, requiring aggressive removal. When this aggressive surgical treatment failed, it was often attributed to the lack of complete cyst and sinus tract removal, inadequate hair removal, or poor hygiene, and subsequent even more aggressive excision was performed. Although recent information discounts the theory of congenital etiology, and an acquired etiology is well accepted [1], the concept of aggressive tissue excision still remains as the most commonly performed operation for pilonidal disease throughout the world [2,3].

We now understand that the true etiology of pilonidal disease is based on the anatomy of a deep gluteal cleft and the subsequent development of midline pits, into which hair penetrate and cause subcutaneous collections of hair, which can cause abscesses or sinus tracts. This understanding makes it possible to logically construct varying different operations to help cure the disease. The different types of procedures for pilonidal disease fall into four distinct groups of commonly performed operations:

1. Excisional procedures including excision with or without primary closure [4,5] and marsupilization [6].

2. Minimally invasive procedures that include pit picking, which removes midline pores and cleans out the cyst [7], Gips procedure which is similar to pit picking but uses a skin trephine to remove the midline pores and sinus tract openings [8], SiLaC, which ablates the sinus with laser-generated heat [9], EPSiT, which cleans out the sinus tract under direct endoscopic vision [10], kshar sutra, which ablates the sinus with chemical impregnated thread [11], phenol injection, which chemically cauterizes the sinus tract [12], and fibrin glue, which obliterates the sinus tract [13].

3. Flap procedures where the incision may cross the midline, such as Z-plasty [14], VY-plasty [15], Limberg flap [16], and rhomboid flap (Dufourmentel flap) [17], which move tissue into the midline to repair defects from tissue removal.

4. Off-midline closure procedures such as the Karydakis flap [18] and the Bascom cleft lift [19], which remove the pilonidal disease, flatten the gluteal cleft, and bring the incision off the midline.

A recent meta-analysis of 6,143 studies by Stauffer et al. has demonstrated the high failure rate of the excisional procedures [20]. The minimally invasive procedures have the advantages of causing minimal tissue disruption and minimal scarring and are easy to perform. However, failure rates, as demonstrated by Stauffer et al., range between 15% and 40% at five years depending on the specific procedure. Not all patients with pilonidal disease are candidates for the minimally invasive procedures.

The other kinds of flap procedures use a different paradigm than the cleft lift. These flap procedures bring tissue into the midline to cover a defect, in contrast to the off-midline closures, which remove tissue in the midline to flatten the cleft.

This article reports a single surgeon's experience with the Bascom cleft lift, which is a rotation flap procedure with an off-midline closure. This was used both as a solution for primary pilonidal disease and as a salvage procedure after the failure of other types of operations including other types of failed flap procedures. The purpose of this study was to report our results with this technique and to determine predictors of success.

## Materials and methods

This series includes all of the author’s cleft lift patients from February 1993 to August 2020. These patients were accrued by normal referral pathways and through internet marketing of a private practice pilonidal clinic. None of the author’s cleft lift patients were excluded from this study. A report on the results of the cleft lift procedure was planned from the start, and therefore data collection began with the first patient. As this experience progressed a significant number of patients were attracted with severe, recurrent, and perianal pilonidal disease, and 80% of the patients were accrued in the last five years of the study. This series of patients includes and augments a series previously reported by this author [21].

Patients who lived locally came to the clinic for evaluation and subsequently had surgery when appropriate for the clinical situation and conducive to the patient's wishes. For patients who were traveling a significant distance, the timing between physical examination and surgery was compressed. The initial contact was an email consultation consisting of a medical history and current photos of the gluteal area. If, on the basis of this information, they were felt to be a candidate for a cleft lift, they traveled to our clinic for a physical examination the day of, or day before, the scheduled operation. Patients were accepted with obvious active disease, such as open wounds, draining sinuses, and painful cysts. But patients were also accepted with histories of abscesses without current active disease or with draining midline pores. If patients had painful fragile scars from previous surgery, which would intermittently open up and heal, they were also considered candidates. The patients were educated about this procedure and alternatives.

Contraindications to surgery were anticoagulation that could not be held during the operative procedure, inability to tolerate anesthesia, severe immobility due to other causes, chronic steroid use, malnutrition, immune suppressants that could not be stopped, and weight over 350 lbs. (159 kg). If patients had unrealistic expectations of the procedure or extreme concern regarding the cosmetic change in the appearance of the buttocks, we suggested that they investigate one of the minimally invasive pilonidal procedures first. Additionally, if a patient not only lived far away from our clinic but was also predicted to have a complex recovery requiring multiple post-operative visits, they were not deemed an appropriate patient for surgery at a location distant from their home, and we suggested they try to find a surgeon locally.

The patients in this series were all operated upon under general anesthesia in the prone position. All patients were discharged the same day as the operation unless there were social issues that made an overnight stay a necessity. Patients who lived a short distance from the clinic usually had two post-operative visits. If patients had traveled a significant distance for surgery, they were asked to remain nearby for between two and seven days for possible post-operative support. Subsequent to that, follow-up visits were virtual, utilizing phone, and digital communication, and usually occurred at weeks 3 and 6 post-operatively.

Data analysis included a Kaplan-Meier curve to estimate recurrence-free survival and predictive models to determine the strongest predictors of recurrence using proportional hazards regression for time-to-event data. The proportional assumption was checked using log-log survival plots and time interactions terms; sex showed non-proportionality and therefore stratified models were used. Each predictor was modeled individually with control for age and sex, and then all predictors were modeled jointly.

The cleft lift procedure

After administration of general anesthesia, the patient is placed prone and flat on the operating table. Intravenous ciprofloxacin and metronidazole, or ampicillin/sulbactam is administered.

**Figure 1:**
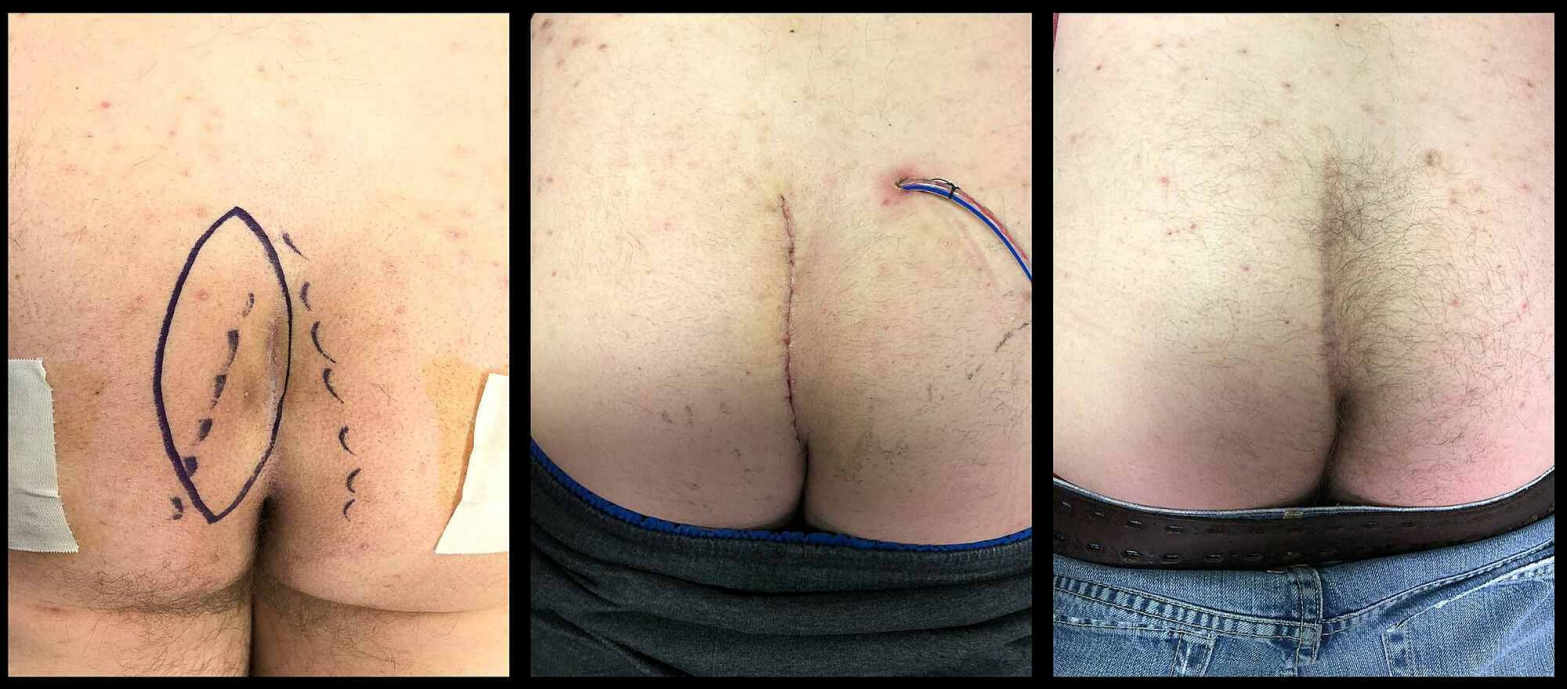
The cleft lift in a patient with multiple previous pilonidal abscesses. The left image shows the elliptical incision, the center image shows the patient at one week, and the right image shows appearance at one year.

The buttocks are compressed medially, and the line of contact between the two sides of the gluteal cleft is marked with a dotted line for future reference (Figure 1). The buttocks are taped apart so that the entire cleft is exposed and the anus is clearly visible. The skin is clipped of hair and marked with the proposed area of excision. No bowel prep was performed nor was the interior of the anal canal examined.

A proposed, vertically oriented, off-center, somewhat elliptical incision is marked such that the entire midline is excised along with any nearby sinus tract openings or masses and the skin on one side of the cleft. This is positioned to remove the side with the most visible disease. The skin is prepped with povidone-iodine antiseptic. The proposed line of incision is infiltrated with 0.5% bupivacaine with epinephrine to minimize bleeding and aid in post-operative pain control.

The medial portion of this elliptical incision is made, and from this cut edge, a skin flap is raised on the side opposite the proposed excision to a thickness of approximately 1 cm and to a width close to the previously marked dotted line. It is reasonable to partially develop the flap in this initial step and subsequently develop it further as needed. A flap that is too wide or too thick may buckle and have negative cosmetic consequences.

Once the flap has been raised, the tapes on the buttocks are removed, and the flap is grasped and brought across the midline. Its ability to reach the previously drawn lateral marking is evaluated, and the mobility of the flap and/or the line of resection is modified as appropriate. This new marking will define the second side of the elliptical incision. In general, removal of 4 cm or more of skin on the side opposite the flap will result in an incision that is satisfactorily away from the midline; less than 4 cm often will not successfully bring the scar far enough.

The second side of the elliptical incision is then made, and the skin on the non-flap side of the cleft along with the midline disease and sinus tracts are removed. Care is taken to remove as little subcutaneous tissue as possible, while removing any sinus tracts, hair, and debris.

The wound is irrigated with sodium oxychlorosene antiseptic solution (Chlorpactin™), and infiltrated with liposomal Marcaine (Exparel™).

The midline subcutaneous fat is sutured together with figure-of-eight sutures of 2-0 poliglecaprone 25 (Monocryl™). Closure of this layer is extremely important as it eliminates dead space, does not allow the skin to fall back in a deep cleft, and assists in lateralization of the incision. The space between the deep closure and the flap is drained with a 15-French channel drain (Blake™) brought out on the upper buttock on the flap side.

The flap is finally sutured in place with interrupted 2-0 poliglecaprone 25 (Monocryl™) sutures in the deeper layers and 3-0 poliglecaprone 25 in the deep dermis with all knots buried. The skin is closed with synthetic absorbable monofilament (4-0 Biosyn™ or Monocryl™) running subcuticular suture. Benzoin and Steri-Strips™ are applied. Figure 1 shows an example of a cleft lift procedure on a patient who had not had previous excisional surgery.

It is imperative that the entire cleft be flattened and the incision is completely off the midline. This can be evaluated during the course of the closure by applying medial pressure on the buttocks and observing the change in configuration that this causes. If the incision dips into a fold and moves to the midline, then more skin on the non-flap side should be removed even if it entails taking the incision apart, resecting more skin, and re-closing the wound.

Modifications in patients with perianal disease

**Figure 2:**
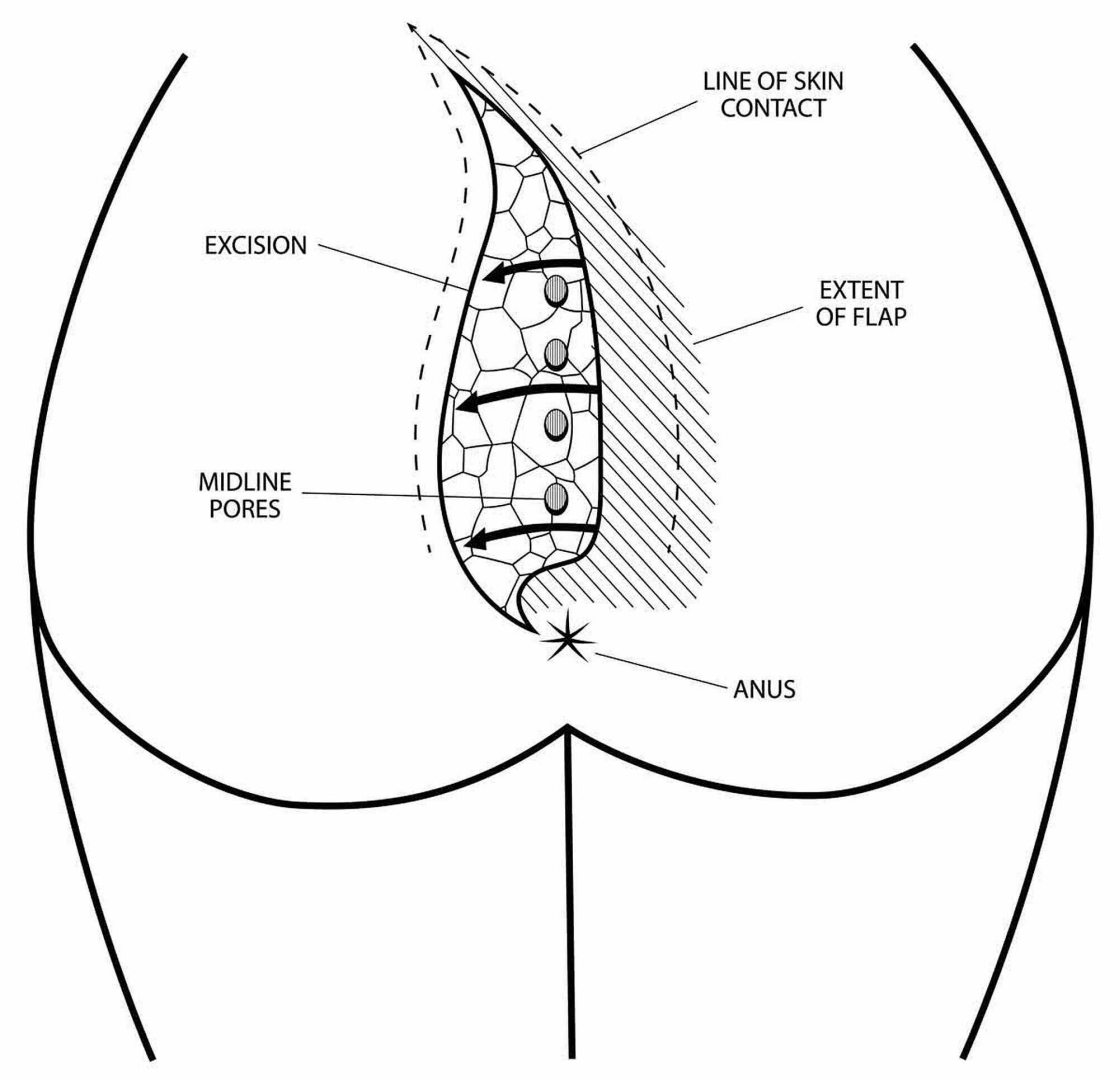
Modifications to the cleft lift procedure for patients with perianal disease

One factor that greatly enhances the difficulty in performing a satisfactory cleft lift is the presence of wounds, pits, or sinus tract openings in proximity to the anus. I define “perianal” as wounds being within 5 cm of the center of the anus; and “edge of anus” disease as a subset of the perianal wounds that actually reach or overlap the anoderm with no normal skin between the wound and the thinned out perianal skin. Some modification in the cleft lift technique was made in order to treat these patients. Figure 2 shows an artist’s rendition of the general concept.

The basic strategy takes advantage of the fact that although the sacral portion of the flap moves laterally from one side to the other, the perianal part of the flap must be an arc rotated downward and laterally, and the incision must curve toward the anus from the side opposite the flap, approaching the anus in the 10 or 11 o’clock position for disease on the left and in the 1 or 2 o'clock position for disease on the right.

It is safe and possible to place the initial medial incision such that it curves below the lowermost opening no matter how close its relationship to the anus.

The dissection always encounters fibers of the superficial anal sphincter, which are left in place. Incontinence has not occurred in this series.

Closure must start at the inferior portion of the incision and gradually proceed superiorly. Suturing the inferior portion of the flap into position must be done such that this medial arc is rotated down in an exaggerated fashion to nestle it into the arc of the lateral limb of the incision.

**Figure 3:**
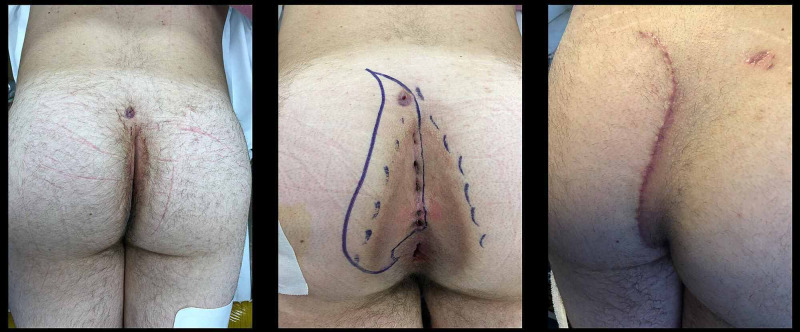
The cleft lift on a patient with perianal disease and a secondary sinus tract opening. The left image is pre-operative, the central center image shows the area for proposed excision, and the right image to the right is six weeks post-operative.

Curving the superior part of the incision away from the midline so that the closed incision is a “lazy S” shape helps equalize length disparities between the two sides of the excision. Figure 3 shows a patient with multiple midline openings and a secondary sinus tract opening before and after a cleft lift. This patient has not had recurrence in over two years.

Post-operative care

The drain is removed sometime after post-operative day 3, as long as the output is less than 20 cc/24 hours. If a patient traveled home to a location that was too far for them to easily return to drain removal, I would have them email me photos of the wound, the drain output record, and a report on their progress. When I gave them approval for drain removal, they had a surgeon, primary care physician, nurse, wound clinic personnel, or family member remove the drain. Patients are asked to avoid sports, biking, or running for six weeks. They are allowed to shower the day after surgery but instructed to avoid soaking in a tub or pool for four weeks. The dressing over the incision and drain site is changed daily for five days. Patients with a perianal limb of the incision are asked to tuck a piece of gauze next to the lower portion of the incision to allow air circulation. This is changed twice daily and continued for four weeks. Ciprofloxacin and metronidazole or ampicillin/clavulanic acid is continued for one week. Patients are encouraged to use acetaminophen or ibuprofen for pain, but an additional narcotic, such as tramadol or hydrocodone, is also prescribed for use if needed. Hair removal is not recommended and has not been utilized in the treatment of these patients. If there are problems with healing of the portion of the incision within 4 cm from the anus, 10% metronidazole ointment is applied twice daily for six weeks, and this has been very successful in promoting healing [22].

Follow-up

Patients were initially followed for at least two months. Patients were surveyed in preparation for this study by phone or email survey in order to determine if there had been recurrence or other problems. Follow-up has been from 2 to 228 months, with 348 followed for over six months, 266 for over 12 months, and 156 for over 24 months. The median follow-up time for patients without recurrence was six months (IQR: 2-19.5). A Kaplan-Meier analysis of the follow-up data was performed, which displays follow-up until the present time until lost to follow-up or until they had a failure or recurrence.

## Results

There were 700 patients in this study (67% male and 33% female). The age range was from 10 to 71 years. Of the 700 patients, 322 (46%) had previous failed surgery and 378 (54%) had not had any previous surgery other than incision and drainage of abscesses. Eight patients (1%) weighed over 300 lbs. (137 kg) (Table 1).

**Table 1:** Patient characteristics

Patient Characteristics	Number	Percent
Mean age	25	
Age range	10-71	
Male	468	67%
Female	232	33%
Weight over 300 lbs. (137 kg)	8	1%
Failed one or more previous operations	322	46%
No previous operations	378	54%

Complications are shown in Table 2. The most common complication was a 2- to 3-mm separation of the lower portion of the wound, which involved the epidermis and dermis, and was at most 2-3 cm in length. This occurred in 10.9% of patients and required no treatment other than having the patient keep it clean and dry. These were all healed within eight weeks from surgery. Wound infections occurred in 2.6% of patients. Two of the patients with a wound infection required wound exploration, irrigation, and drain replacement. The rest were treated with antibiotics, and a delay in drain removal if the drain was still in place. We considered any degree of cellulitis that required additional antibiotics beyond the first week a wound infection.

**Table 2:** Complications in 700 patients

Complication	Number	Percent
Slight wound separation	76	10.9%
Wound infection	18	2.6%
Hematoma	8	1.1%
Wound dehiscence	5	0.7%

Post-operative hematomas occurred in 1.1% of patients and were usually associated with a post-operative fall, anticoagulation, or an undiagnosed bleeding disorder. Aside from one patient who fell after he restarted Lovenox® post-operatively, these were treated non-operatively by leaving the suction drain in place until there was no more bloody drainage.

Wound dehiscence occurred very rarely and was seen in five (0.7%) patients. It was associated with trauma, poor wound care, technical error, or a persistent seroma after the drain came out. It was treated by re-suturing the wound or revising the shape of the flap. If a revision of the flap was required, these patients were included in the “required surgical revision” group. There were no other seromas in this series of patients.

There were no cases of osteomyelitis, fecal incontinence, inadvertent entry into the anal canal during surgery, fistulization to the rectum from advanced disease, flap necrosis, or wound dehiscence caused by tension.

**Figure 4:**
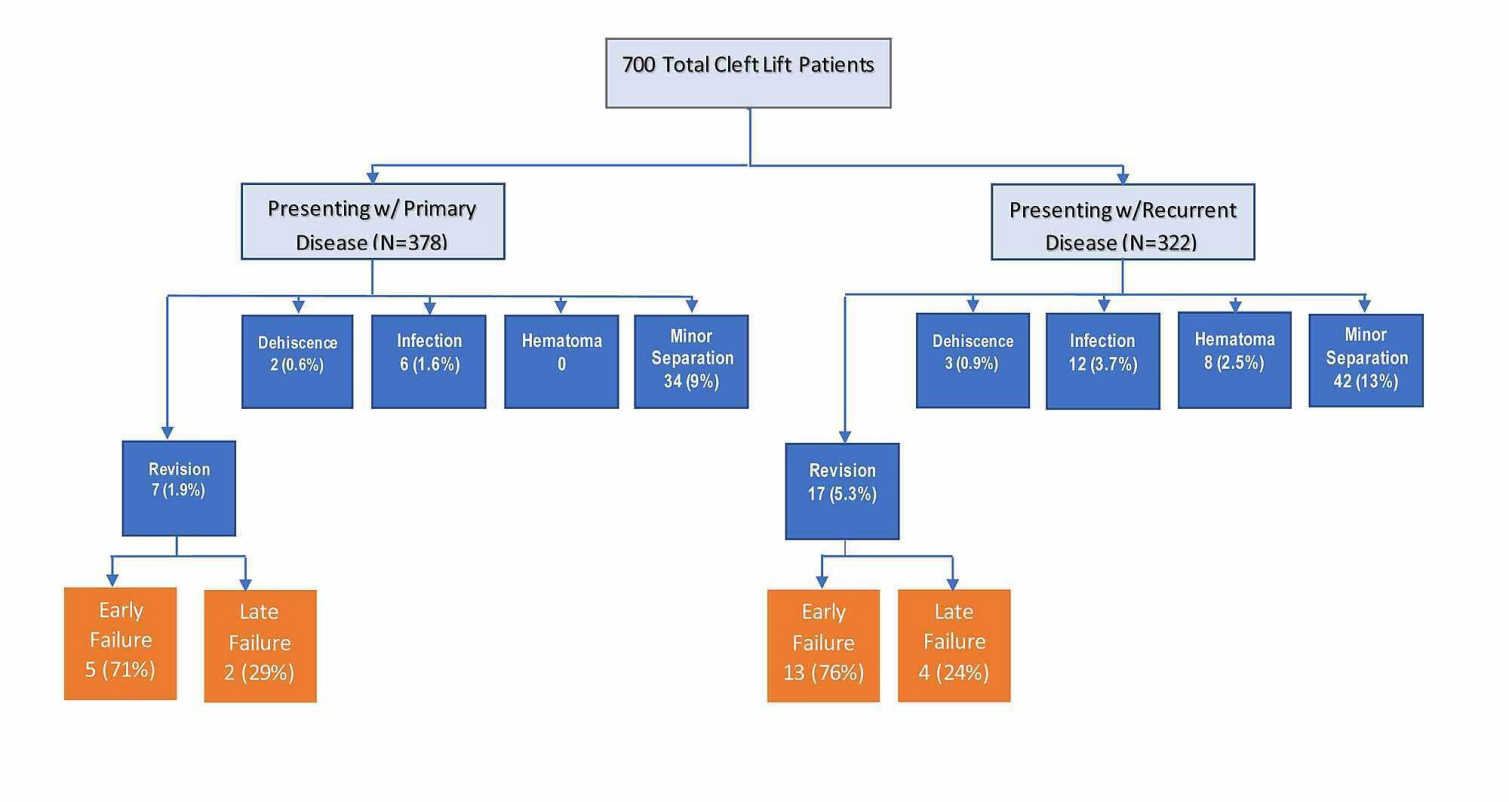
Complications related to primary operation vs. recurrent disease Early failure ≤ six months and late failure > six months.

Figure 4 shows the complication rates as they related to whether the patient presented with primary disease or had previous failed operations. Each type of complication occurred more frequently in the previously operated patient. Evidence of failure, either by an incisional complication or a recurrent sinus tract, occurred more frequently within the first six post-operative months than after six months, with a ratio of approximately 3:1.

The overall rate of revision was 3.4% (95% CI: 2.1-4.8) and was used as a measure of the success or failure of the procedure (Table 3). If a patient had a part of the incision that did not heal, it was considered a failure. Breakdown of the incision or development of a recurrent sinus or abscess after initial healing was considered a recurrence. There were no failures or recurrences that were not treated by revision. The cumulative failure and recurrence-free rate was 96.2% (95% Cl: 0.945-0.98) at six months, 95.6% (95% Cl: 0.937-0.976) at one year, and 93.6% (95% Cl: 0.908-0.964) at two years.

**Table 3:** Revision rate by category of disease *This includes patients who previously had a Z-plasty, Limberg flap, rhomboid (Dufourmentel) flap, gluteal flap, VY-plasty, or cleft lift at the time they presented to the author.

Category of Disease	Total	Revisions	Percent
No previous surgery	378	7	1.9%
Previous surgery	322	17	5.3%
Previous flap surgery*	62	3	7.1%
Without perianal disease	462	13	2.8%
Perianal disease	238	11	4.6%
Without either perianal disease or previous surgery	340	5	1.3%
With both perianal disease and previous surgery	200	9	4.5%
Perianal disease not at the edge of the anus	192	4	2.1%
Edge of anus disease	45	7	15.6%
Total patients in the study	700	24	3.4%

Wounds actually on the anoderm (“edge of anus” wounds) had the highest failure rate and required significant technical modifications in order to obtain success. In this series, 34% of patients fell into the “perianal” category with 19% of these being “edge of anus”. Although disease in this location is unusual, we had 45 such patients in this series because we attracted patients with recurrences and failures. The initial cleft lift was successful 84.4% of the time in these patients, i.e., 38 out 45 were successful. Of the remaining seven patients, all but one was successfully repaired by revision of the cleft lift. 

Failure rates in patients who had previous surgery was 5.3%, perianal wounds not at the edge of the anus was 2.1%, non-perianal disease was 2.8%, or no previous surgery and non-perianal location was 1.3%. In both individual and joint statistical models, predictors of whether revision would be required were (1) whether the operation was primary or for salvage or (2) whether a wound extended to the edge of the anus. Having a perianal wound that did not extend to the edge of the anus or having previous failed flap surgery did not predict revision in either set of models. Rate of revision was almost three times as high in salvage cases as primary cases (adjusted hazard ratio [aHR]: 2.97; 95% CI: 1.11-7.95); and more than six times as high in those with a wound to the edge of the anus as compared to those without (aHR: 6.20; 95% CI: 1.88-20.48). None of the patients over 300 lbs. (137 kg) required revision.

**Figure 5:**
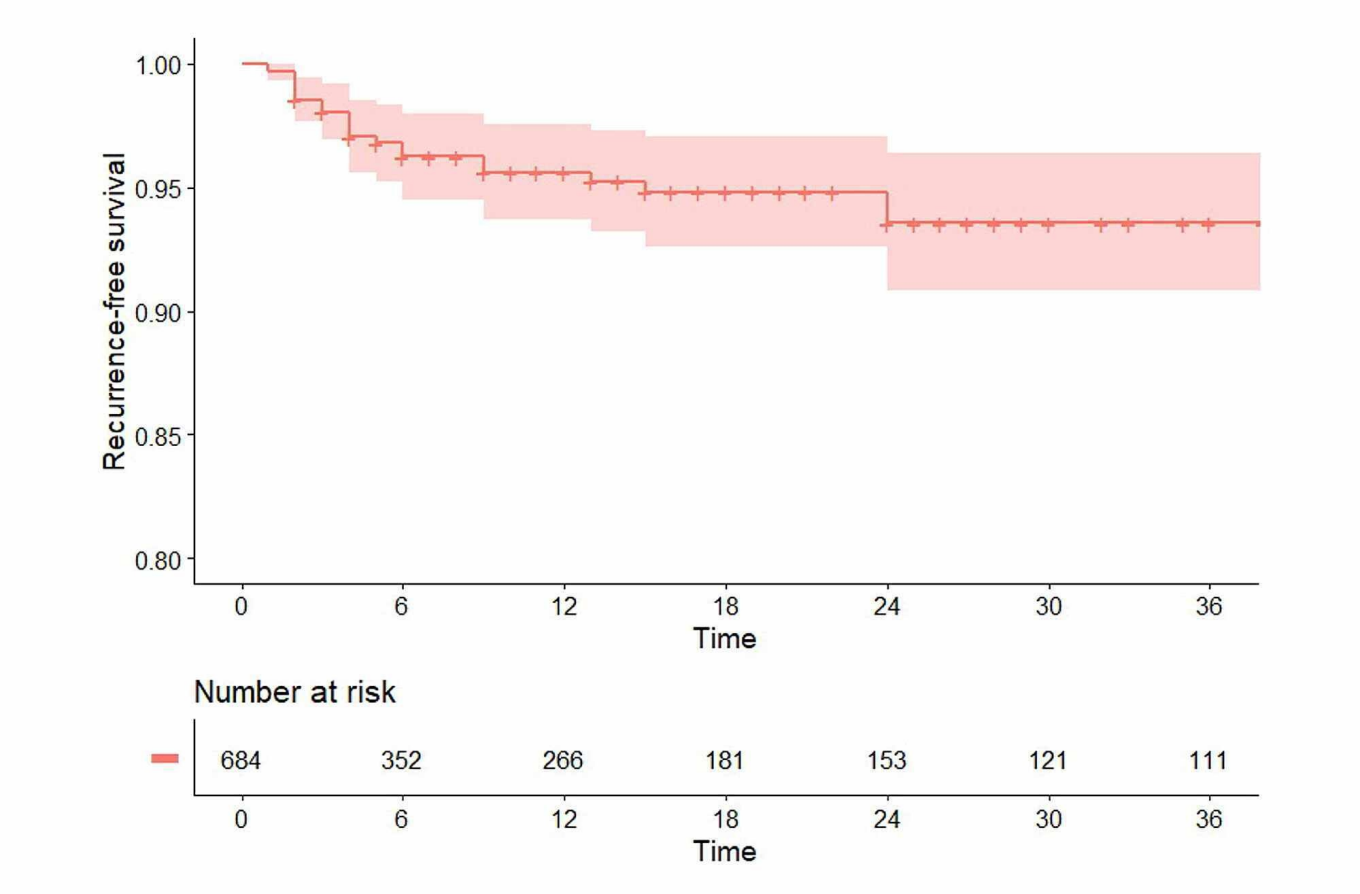
Cumulative recurrence-free rates for 700 patients who were treated with the cleft lift procedure between 1993 and 2020.

A Kaplan-Meier analysis of the revision rate (failure), as shown in Figure 5, demonstrates that both recurrences and failures are uncommon and occur early and that there were no recurrences after patients passed the 24-month time frame. The survival estimate at t = 24 (when the last event occurs) was 0.93 (95% CI: 0.90-0.96). The median follow-up time for patients without recurrence was six months (IQR: 2-20). This analysis assumes that the dropouts are not much different from the completers, which is appropriate since most of the recurrences occurred early in the follow-up period when patients are most apt to return to their original surgeon for correction

**Figure 6:**
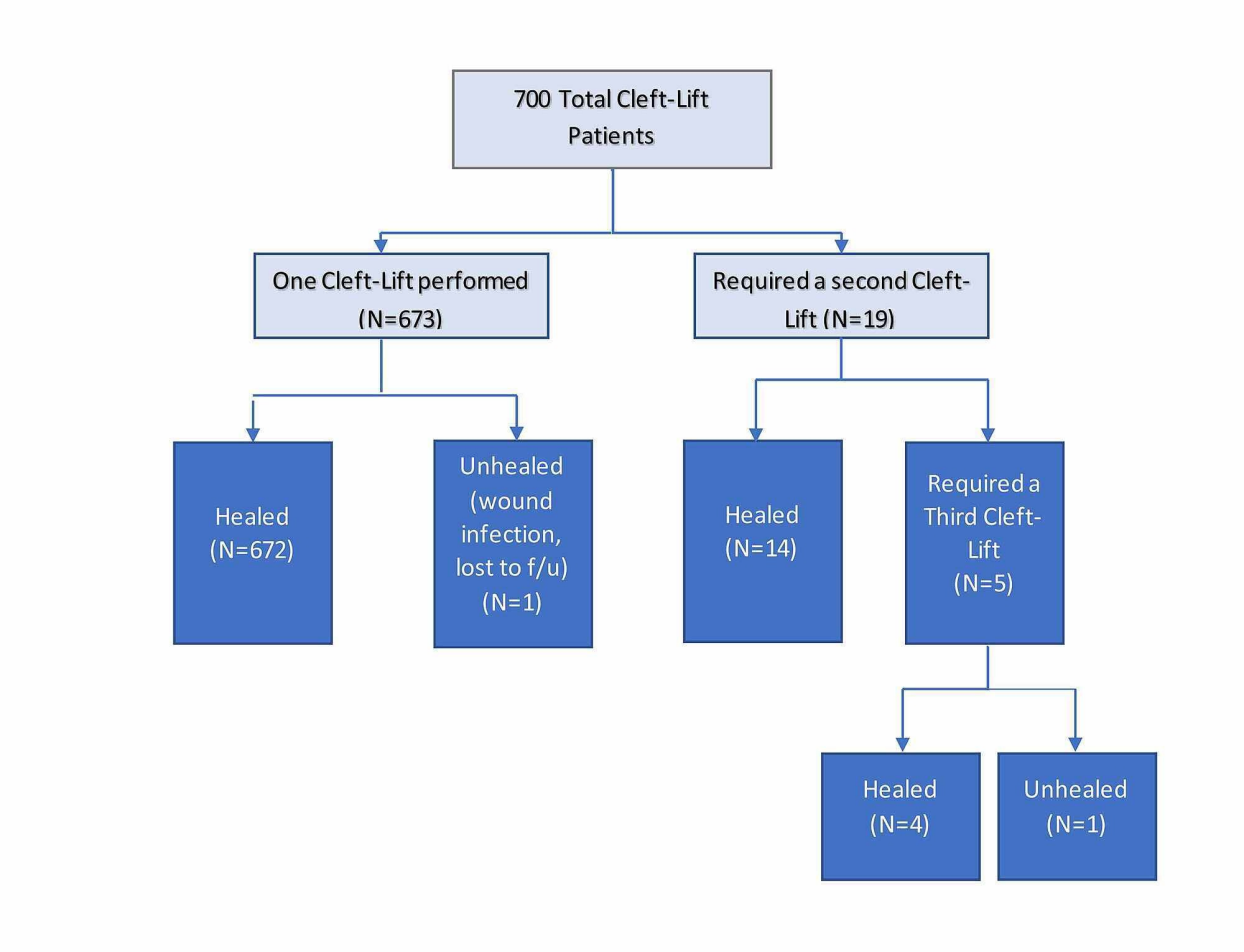
Number of procedures required and healed status of 700 patients.

Revisions were usually successful and consisted of bringing the flap farther away from the midline and flattening the cleft even lower. In this series of673 patients, 96.1% were cured with a single cleft-lift procedure, 19 (2.7%) required two procedures, and 5 (0.7%) required three procedures. Overall, 698 (99.7%) patients were successfully healed. Two patients still have wounds that have not yet healed (0.3%). This is demonstrated in Figure 6.

## Discussion

This series of patients demonstrates that excellent results (96.1% healed after one cleft lift) can be obtained with the Bascom cleft lift when caring for patients with primary pilonidal disease, failed excisional surgery, failed flap surgery, and extremely low perianal disease with “edge of anus” wounds. We did not find a category of patients in which high success rates could not be obtained. It should be emphasized that this is an unusual group of patients, with 46% presenting after failing one or more previous operations, i.e., far more advanced disease than would routinely be encountered in a surgical practice. In this study, we consider both early failures and late recurrences to be manifestations of the same problem, i.e., failure to reconstruct the gluteal cleft such that it has been adequately flattened and the incision placed off-midline. For this reason, we view the revision rate to be an excellent measure of the success of the procedure whether failures occur early or late.

The key technical points are that it is important to flatten the gluteal cleft throughout its entire length, position the scar off the midline, and remove as little subcutaneous tissue as possible. Because this is primarily a disease of the skin, aggressive removal of subcutaneous tissue is unnecessary and can be harmful. Although the steps to perform this operation are straightforward, there is a degree of subjectivity and uncertainty in deciding when the cleft has been flattened enough and the scar is situated far enough off the midline. Success improves with mentoring and experience, and early failures in a surgeon's experience should be analyzed as part of the learning process and not viewed as an indictment of the operation itself.

As we cared for these patients, it became clear that they fell into several categories. The particular situations that impacted the success rate and difficulty of the procedure were previous failed surgery, perianal disease, and the more extreme version of perianal disease, i.e., “edge of anus” disease. A specific category of patients with previous surgery include those with failed flap procedures of various kinds (VY-plasty, Z-plasty, Limberg flap, rhomboid flap, cleft lift, Karydakis flap, gluteal flap, etc.), and they present their own unique challenges in order to maintain blood supply to the previously rearranged tissue, but the basic concepts of the cleft lift are still applicable.

The recovery for a cleft lift is usually very straightforward and is conducive to treating patients who travel a distance for care. There are no complex and prolonged dressings nor any need for repeated surgical clinic visits, home-care nursing, or wound clinic referral, and the failure rate is quite low.

Although it was not possible to obtain follow-up on all patients from 1993 until 2020, this study includes all patients followed by the author until complete healing, and many followed for longer than two years. The fact that this study encompasses a surgeon’s entire experience from initially learning the procedure to becoming very experienced has value in showing that a surgeon can successfully integrate this technique into their practice. The success of this operation in our clinic is not unique, and there are other reports of similar data [23-25]. Hopefully, surgeons will consider adding this technique to their repertoire of operations for pilonidal disease. This was the first operation for pilonidal disease (excluding incision and drainage procedures) for many of these patients, and it worked very well at removing their current disease and preventing recurrences. This procedure changes the appearance of the gluteal area and does leave a scar of significant length; therefore, patients and surgeons have to consider these factors when choosing an operation. Many of the patients in this series specifically sought cleft lift surgery as their first operation and traveled a great distance to have it performed. Additionally, the cleft lift procedure is an excellent solution for the difficult situations that come up in pilonidal patients with perianal and recurrent disease. Many of these patients had endured repeated, failed, wide excisions. With each failure, the resulting scarring and wounds appeared more inferiorly in the gluteal crease. Once the wounds reached the anoderm, they were often declared irreparable by the original surgeon, and indeed the literature gives little assistance in providing a solution for pilonidal wounds on the anus. This study describes a strategy that is quite effective when treating "edge of anus" wounds.

There are other excellent techniques for treating pilonidal disease, particularly the “minimally invasive” techniques. These do not have a success rate as high as the Bascom cleft lift and are not applicable to patients with open wounds, but their less invasive nature makes them appealing to patients and surgeons. This study is limited to evaluating the cleft lift as treatment in all patients. Future research to determine how to optimally integrate the minimally invasive procedures with the cleft lift operation will be very valuable.

A complete discussion of this disease would be incomplete if it did not address the psychological toll that this takes on patients. Most of these patients are in their teens and twenties and possibly have never had to interface with the healthcare system for any serious problem before they developed pilonidal disease. Because of the high failure rate of the excisional procedures coupled with the painful and burdensome process of healing by secondary intention, they often view their first operation as a highly traumatic and unsuccessful ordeal. We have seen numerous patients who have had multiple failed operations (as many as 15) and have had years of their life consumed by medical care. Many have lost employment, romantic relationships, and educational and sports opportunities in these formative years of their lives. They are severely traumatized by this, and we often see signs of post-traumatic stress and depression in these young patients. Lack of activity contributes to weight gain, and many of these patients have become obese during this process. Two years of wound care and repeated operations is not at all unusual, and many have endured five or more years. Once they have had successful surgery, it often takes them many months to accept that it is repaired and that they can get on with their lives. Because of the high failure rate and prolonged and difficult recovery associated with the excisional procedures, it can be argued that they should only be used when other more sophisticated procedures are not available.

## Conclusions

The cleft lift is an excellent operation for pilonidal disease. It can be performed in the primary patient without perianal disease, with a 98.7% success rate. With an overall success rate of 96.6%, it is an extremely effective option for patients with complex and recurrent disease. Although the subset of patients with wounds on the edge of the anus have a higher failure rate than other groups, there are few other options available to them with a success rate this high, and they are considered good candidates for this procedure.
